# Ninety-day all-cause emergency room use among coronary artery bypass grafting patients associated with near-infrared fluorescence imaging: a retrospective cohort study

**DOI:** 10.1097/MS9.0000000000000206

**Published:** 2023-02-07

**Authors:** Michelle P. Sosa, Deirdre G. McNicholas, Arbelina B. Bebla, Seth Emont, Zhun Cao, Craig Lipkin, Vivek Ajmani, Derek D. Muehrcke

**Affiliations:** a Stryker Corporation, San Jose, California; b Premier Applied Sciences, Premier Inc., Charlotte, North Carolina; c Cardiothoracic & Vascular Surgical Associates, St. Augustine, Florida, USA

**Keywords:** coronary artery bypass grafting, emergency room, indocyanine green, intraoperative graft patency assessment, near-infrared fluorescence imaging

## Abstract

Reducing emergency room (ER) use may indicate the improved quality of patient care at index hospitalization. The aim of this study is to determine whether the use of near-infrared fluorescence (NIRF) imaging with indocyanine green (ICG) during coronary artery bypass grafting (CABG) surgery is associated with a lowered 90-day all-cause ER use.

**Materials and Methods:**

This retrospective cohort study included adult patients with inpatient hospitalizations between January 2016 and June 2020 for an isolated CABG procedure at a US hospital. Propensity score matching was used to create matched cohorts to address the differences in patient, payer type, hospital, and clinical characteristics. A multivariable regression analysis was conducted to determine the association of NIRF imaging with ICG on ER use within 90 days of discharge after controlling for patient, payer type, hospital, and clinical covariates.

**Results:**

In total, 230 506 adult patients underwent an isolated CABG procedure. Less than 1% (n=1965) were assessed with NIRF imaging using ICG. There were differences in patient demographic and hospital characteristics between the treatment group (i.e. NIRF with ICG) and the comparison group (i.e. no NIRF with ICG). After controlling for covariates, a statistically significant lower 90-day all-cause ER use was documented among the treatment group (adjusted odds ratio=0.84, 95% confidence interval=0.73–0.96, *P*<0.009). Reasons associated with ER use were similar between the two groups.

**Conclusion:**

Routine intraoperative graft patency assessment with NIRF imaging using ICG may help to improve a patient’s care experience and reduce subsequent resource utilization. Intraoperative graft patency assessment with NIRF imaging using ICG is associated with a 90-day all-cause ER use reduction among CABG patients. Further studies are needed to compare the ER usage among centers that used this technique versus those that did not to determine if associated reductions in ER use are a center or technique-specific phenomenon.

Highlights

- NIRF with ICG is a quality assurance adjuvant for graft evaluation during coronary artery bypass grafting (CABG).
- Reduced emergency room (ER) use may indicate improved quality of care at index hospitalization.
- NIRF with ICG patients had lower 90-day ER use than no near-infrared fluorescence (NIRF) with indocyanine green (ICG) patients.
- NIRF with ICG may improve a patient’s care experience by reducing ER usage.

## Introduction

Graft patency is a significant determinant of long-term prognosis after CABG surgery^1^. Early graft failures are predominantly attributed to technical anastomotic problems and may be corrected if identified intraoperatively^2^.

**Figure 1:**
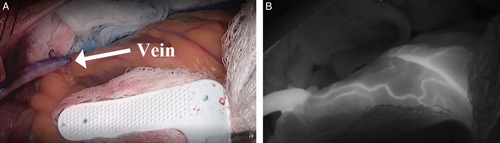
Real-time assessment of distal myocardial perfusion and patency of the vein graft using intraoperative NIRF imaging with ICG. ICG is injected directly down the vein by the surgeon to evaluate distal myocardial perfusion and patency of the vein graft (white arrow) in real time. The fluorescence image is displayed in grayscale with ICG fluorescence indicated by the color white. All other areas of the image appear dark. (A) Without NIRF with ICG. (B) With NIRF with ICG. ICG, indocyanine green; NIRF, near-infrared fluorescence.

Studies demonstrate that NIRF imaging using ICG, also referred to as intraoperative fluorescence imaging (IFI), reliably assesses coronary artery bypass graft patency^2^–^7^, while also reducing hospital cost of CABG^8^,^9^. IFI is based on the fluorescence properties of ICG dye and enables cardiac surgeons to visualize blood flow in native coronary arteries, bypass grafts, and myocardial distribution during the procedure. After intravenous injection, ICG binds immediately to plasma proteins and travels through the circulatory system before being rapidly metabolized by the liver and subsequently secreted into bile. When the protein-bound ICG is excited by near-infrared light, it generates fluorescence that is then captured as it flows through the graft. The fluorescence image is displayed in grayscale with ICG fluorescence indicated by the color white and all other areas of the image appearing dark (Fig. 1). IFI’s ability to help identify adequate perfusion in bypass grafts makes possible early detection and immediate correction of graft failure if required, which may help reduce procedure-related early graft failures in CABG patients^5^. This is significant because perioperative graft failure following CABG may lead to morbidities such as acute myocardial ischemia^10^ and higher in-hospital mortality^11^ among other adverse cardiac events. IFI is used to enhance visual assessment in numerous surgical indications,^12^–^15^ as it is safe, relatively inexpensive, easy to perform, and repeatable.

There is a growing interest in the postindex hospitalization healthcare resource utilization of CABG patients. Current efforts to improve healthcare focus on hospital readmissions, but ER use is also essential considering CABG patients frequently require hospital-based, acute care postindex hospitalization^16^,^17^, Approximately 15% of patients are readmitted within 30 days after being discharged from an isolated CABG surgery, while nearly 12% return to the ER^16^. A return to the ER often represents a clinically meaningful event for patients, increases cost of care, and visitation frequency may be inversely related to the quality of care received^16^. Therefore, reduced ER use may indicate an improvement in the quality of patient care at index hospitalization.

Because IFI helps to intraoperatively detect deficit grafts which might otherwise be overlooked and not revised at the initial CABG procedure^2^,^3^, its use during CABG may help to reduce subsequent resource utilization postindex hospitalization. While most quality metric systems are interested in 30-day postoperative outcomes, there is growing interest in 90-day postoperative outcomes as well^18^,^19^. The aim of this study is to determine if intraoperative assessment with IFI may be associated with 90-day all-cause ER use following CABG surgery. This study examines the differences in ER use within 90 days of discharge of patients undergoing CABG procedures with and without IFI.

## Materials and methods

### Study design

A retrospective cohort study design was used to analyze the patient demographics, payer type, hospital characteristics, clinical characteristics, and medical resource utilization of patients who underwent isolated CABG procedures and were discharged from US hospitals between 1 January 2016 and 30 June 2020. The design of the study is summarized in the Graphical Abstract. This paper was retrospectively registered in Research Registry (researchregistry8521). Also, this work has been reported in line with the STROCSS criteria^20^ (Supplemental Digital Content 1, http://links.lww.com/MS9/A14).

### Data source

We utilized hospital administrative data from the all-payer PINC AI Healthcare Database (formerly Premier Healthcare Database; Premier Applied Sciences, Premier Inc., Charlotte, North Carolina, USA). The PINC AI is a hospital-based, service-level, all-payer database containing discharge information from geographically diverse hospitals and their inpatient and hospital-based outpatient visits^21^. This data has been used in numerous publications^22^–^25^ and represents more than 1.1 billion patient encounters, equating to approximately one in every five inpatient discharges in the USA. The data come from standard hospital discharge files including patient demographics, disease states, and information on billed services in deidentified patient daily service records. All data were statistically deidentified, and results were presented in an aggregated fashion. Due to Premier’s rigorous quality assurance and billing reconciliation program, less than 1% of patient records have missing information. Based on US Title 45 Code of Federal Regulations, Part 46, the study was exempt from institutional review board approval. The study consisted of records-based research and as such was exempt from ethics committee review and was Health Insurance Portability and Accountability Act-compliant. In addition, the PINC AI includes hospital characteristics data from participating hospitals in its healthcare alliance, including geographic location, population served, bed capacity, and teaching status.

### Exposure variable

The primary exposure of this study is the use of NIRF imaging with ICG during isolated CABG surgery. Patients receiving NIRF imaging with ICG were assigned to the treatment group, and patients without evidence of NIRF imaging with ICG served as the comparison group. Chargemaster descriptions including National Drug Code identifiers and ICD-10-PCS 4A12XSH code for “Monitoring of Cardiac Vascular Perfusion using Indocyanine Green Dye, External Approach” were used to identify patients who received NIRF imaging technology and ICG (Table 1).

**Table 1:** Codes and chargemaster text strings used to identify patients assessed with intraoperative near-infrared imaging using indocyanine green

Identifier Type	Identifier
ICD-10-PCS Code	4A12XSH (Monitoring of Cardiac Vascular Perfusion using Indocyanine Green Dye, External Approach)
Chargemaster text strings	SPY SPY Elite SPY-PHI SPY PHI SPYPHI Fluorescence Fluorescence Vascular Angiography Fluorescent Vascular Angiography Indocyanine Green ICG Green Dye Near infrared fluorescence Imaging Near-infrared fluorescence Imaging NIRF NIF
National Drug Code	66259-0306-13 (SPY Elite Kit: 1 kit in 1 KIT×10 ml in 1 vial, plastic×1 injection, powder, lyophilized, for solution in 1 vial (66259-146-01) 66259-0306-23 (SPY Elite Kit: 6 kit in 1 case >1 kit in 1 kit×10 ml in 1 vial, plastic×1 injection, powder, lyophilized, for solution in 1 vial (66259-146-01) 66259-0903-14 (SPY-PHI Kit: Package Description: 1 kit in 1 KIT×1 injection, powder, lyophilized, for solution in 1 vial (66259-146-01)×10 ml in 1 vial, plastic) 66259-0903-24 (SPY-PHI Pack - 6 SPY-PHI Kits: Package Description: 6 KIT in 1 case > 1 kit in 1 kit×1 injection, powder, lyophilized, for solution in 1 vial (66259-146-01)×10 ml in 1 vial, plastic) 49673-0424-01 (Indocyanine Green: 1 injection, powder, lyophilized, for solution in 1 vial, glass) 66259-0146-01 (SPY Agent Green) 49673-0424-01 (Akorn Inc Indocyanine Green: IC-Green Indocyanine Green 25 mg Intravenous Injection Single Dose Vial 6 Vials) 70100-0424-02 (Diagnostic Green Gmbh Indocyanine Green: 6 package in 1 carton>1 kit in 1 package×10 ml in 1 vial, plastic (0409-4887-17)×1 injection, powder, lyophilized, for solution in 1 vial)

ICD-10-PCS, *International Classification of Diseases, 10th Revision, Procedure Coding System*; ICG, indocyanine green; NIF, near-infrared fluorescence; NIRF, near-infrared fluorescence; SPY-PHI, SPY Portable Handheld Imager.

### Inclusion and exclusion criteria

Data on patients 18 years or older with an ICD-10-CM/PCS or MS-DRG code for an isolated CABG procedure without percutaneous transluminal coronary angioplasty (DRG 233, 234, 235, 236) with an index admission between 1 January 2016 and 30 June 2020 were included in the study.

**Figure 2:**
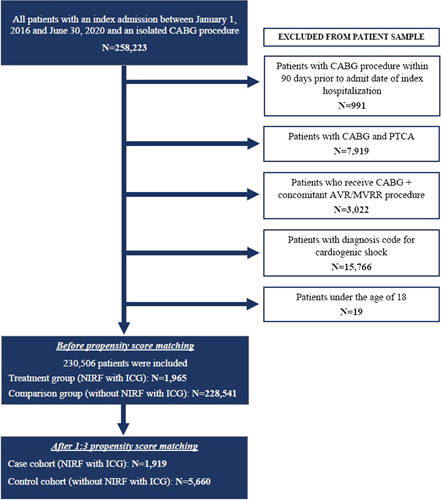
Patient attrition flowchart with inclusion and exclusion criteria showing how many patients were included in the study. AVR, aortic valve replacement; CABG, coronary artery bypass grafting; ICG, indocyanine green; MVRR, mitral valve replacement/repair; NIRF, near-infrared fluorescence; PTCA, percutaneous transluminal coronary angioplasty.

Patients were excluded if they had a CABG procedure within 90 days before their index hospitalization admission date. Patients with evidence of CABG and percutaneous transluminal coronary angioplasty or CABG and concomitant aortic valve replacement or CABG and mitral valve replacement or repair or CABG and transcatheter aortic valve replacement or implantation or CABG and cardiogenic shock were also excluded. The patient attrition flowchart with the inclusion and exclusion criteria is summarized in Figure 2.

### Outcome variables

The primary outcome was ER use associated with the hospital visit within 90 days following index discharge for CABG surgery.

### Statistical analysis

Descriptive statistics were used to characterize and compare baseline demographics, payer, hospital, and clinical characteristics, and healthcare resource utilization outcomes at 90 days postindex discharge. Data measured on a continuous scale are expressed as mean, SD, median, and interquartile range. Categorical data are expressed as counts and percentages of patients in the various categories. Variance inflation factors were calculated for all predictor variables to assess potential multicollinearity for the regression analysis. Initially, two variables, risk of mortality and severity of illness, were found to be correlated with patient comorbidities, resulting in variance inflation factor values more than 5. We chose to retain the patient comorbidities measure (Charlson Comorbidity Index) since it predicts not only greater mortality risk but also more severe comorbid conditions. After removal of the risk of mortality and severity of illness measures, all variance inflation factor values were less than 3, which is an acceptable threshold suggesting low variance inflation.

Propensity score matching was used to create matched cohorts to address the differences in patient, payer type, hospital, and clinical characteristics. Each patient in the NIRF imaging with ICG cohort (case-cohort) was matched to three patients in the without NIRF imaging with ICG cohort (control cohort). Candidate covariates for pair matching included sex, age, race, ethnicity, payer type, hospital size, hospital teaching status, population served (i.e. urban vs. rural), geographic location of hospital, patient medical comorbidities, presence of obesity, use of multiple grafts during CABG procedure, use of ultrasonography (ICD-10-PCS codes B240YZZ, B240ZZ3, B240ZZ4, B240ZZZ, B241YZZ, B241ZZ3, B241ZZ4, and B241ZZZ) as a proxy to capture use of transit time flow measurement (TTFM) during CABG procedure, and use of robotic-assisted surgery (ICD-10-PCS codes 8E0W0CZ, 8E0W3CZ, 8E0W4CZ, 8E0W7CZ, 8E0W8CZ, and 8E0WXCZ) during CABG procedure.

Pair matching without replacement was performed using nearest neighbor matching within a specified caliper distance. Control patients whose propensity score was within the caliper (defined as 0.2 of the SD of the logit of the propensity score) were identified as initial matched candidates since this has been shown to eliminate 99% of bias due to the measured confounders^26^,^27^. Propensity scores were estimated using a generalized linear model as the distance measure and logistic regression (i.e. logit) as the link argument. Covariate balance was assessed prematching and postmatching by calculating standardized mean differences and empirical cumulative density function, where standardized mean differences and empirical cumulative density function values approaching zero indicate balance has been achieved (with values less than 0.1 indicating negligible differences in covariate means or prevalence between the two groups). Visual diagnostics were used to inspect the matching process, including Q–Q (quantile–quantile) and jitter plots (data only shown for standardized mean differences).

Once an acceptable balance was achieved, logistic regression analysis was conducted to determine the association of procedure type (i.e. CABG surgery with and without NIRF imaging with ICG) to 90-day ER use after controlling for other predictors of healthcare utilization, including patient demographic and clinical characteristics and hospital characteristics. The treatment effect was estimated using a standard regression function (generalized linear model) that incorporated covariate adjustment. In addition, cluster-robust SEs were used to account for dependence between observations within the matched pairs. Bootstrapping with 2999 replications was also used to estimate the main treatment effect of NIRF imaging with ICG. Bootstrap confidence intervals were calculated using the bias-corrected accelerated bootstrap method. The same variables used for matching also were used to predict 90-day ER use. We set confidence intervals at 95% and considered a *P*-value less than 0.05 as statistically significant. All analyses were performed using SAS 9.4 software (SAS Institute Inc., Cary, North Carolina, USA) and R Studio (Rstudio: Integrated Development Environment for R; Rstudio, PBC, Boston, Massachusetts, USA).

## Results

### Baseline characteristics

Eighty-nine percent (n=230 506) of the 258 223 patients who underwent an isolated CABG procedure between 1 January 2016 and 30 June 2020 were included in the study. Of these, less than 1% (n=1965) were in the treatment group (i.e. NIRF with ICG) and 99% (n=228 541) were in the comparison group (i.e. without NIRF with ICG) (Fig. 2).

The unmatched NIRF with ICG group had a higher proportion of Hispanic or Latino patients and a lower proportion of not Hispanic or Latino patients than the comparison group (Table 2). A higher proportion of patients treated with NIRF with ICG were seen in smaller capacity hospitals (1–299 beds) than the comparison group. The treatment group also had a lower proportion of patients receive NIRF with ICG in hospitals in the Northeast or Midwest compared with patients who were not treated with NIRF with ICG (Table 2). Following the matching process and based on the absolute standardized mean differences meeting the less than 0.1 threshold, pair matching was achieved across all predictor variables.

**Table 2:** Baseline patient demographics of the study population stratified by treatment status

	Before propensity score matching				After propensity score matching			
Characteristics	NIRF with ICG (N=1965) [n (%)]	Comparison group (N=228 541) [n (%)]	ASMD	*P*	NIRF with ICG (N=1919) [n (%)]	Comparison group (N=5660) [n (%)]	ASMD	*P*
Patient demographics								
Sex								
Female	483 (24.6)	56 794 (24.9)	0.002	0.782	468 (24.4)	1392 (24.6)	0.004	0.856
Age (years)								
Mean (SD)	66.1 (10.2)	66.1 (10.0)	0.005	0.985	66.0 (10.2)	66.0 (9.9)	0.009	0.896
Median (IQR)	67.0 (59.0, 73.0)	67.0 (60.0, 73.0)			66.0 (59.0, 73.0)	67.0 (59.0, 73.0)		
Race								
White	1703 (86.7)	187 068 (81.9)	0.050	<0.0001	1668 (86.9)	4916 (86.9)	0.000	0.958
Black	92 (4.7)	15 256 (6.7)	0.020		88 (4.6)	257 (4.5)	0.001	
Asian	37 (1.9)	4941 (2.2)	0.003		36 (1.9)	98 (1.7)	0.002	
Other/unknown	133 (6.8)	21276 (9.3)	0.027		127 (6.6)	389 (6.9)	0.003	
Ethnicity								
Hispanic or Latino	437 (22.2)	13 387 (5.9)	0.164	<0.0001	424 (22.1)	1271 (22.5)	0.014	0.712
Not Hispanic or Latino	1040 (52.9)	170 552 (74.6)	0.219		1013 (52.8)	3020 (53.4)	0.002	
Unknown	488 (24.8)	44 602 (19.5)	0.055		482 (25.1)	1369 (24.2)	0.012	
Payer type								
Medicare	1158 (58.9)	133 300 (58.3)	0.003	<0.0001	1121 (58.4)	3344 (59.1)	0.003	0.943
Medicaid	140 (7.1)	17 355 (7.6)	0.004		137 (7.1)	420 (7.4)	0.003	
Commercial indemnity	79 (4.0)	15 701 (6.9)	0.029		78 (4.1)	216 (3.8)	0.003	
Commercial managed care	438 (22.3)	47 415 (20.7)	0.017		433 (22.6)	1225 (21.6)	0.006	
Charity/indigent	34 (1.7)	958 (0.4)	0.014		34 (1.8)	98 (1.7)	0.000	
Other*	116 (5.9)	13 812 (6.0)	0.000		116 (6.0)	357 (6.3)	0.004	
Hospital characteristics								
Hospital size (# of beds)								
1−299 beds	647 (32.9)	40 063 (17.5)	0.153	<0.0001	632 (32.9)	2020 (35.7)	0.023	0.020
300−499 beds	660 (33.6)	74 335 (32.5)	0.010		642 (33.5)	1714 (30.3)	0.023	
500+ beds	658 (33.5)	114 143 (49.9)	0.164		645 (33.6)	1926 (34.0)	0.001	
Teaching status								
Nonteaching hospital	933 (47.5)	95 150 (41.6)	0.059	<0.0001	914 (47.6)	2727 (48.2)	0.002	0.676
Teaching hospital	1032 (52.5)	133 391 (58.4)			1005 (52.4)	2933 (51.8)		
Population served								
Rural	25 (1.3)	18 830 (8.2)	0.069	<0.0001	25 (1.3)	90 (1.6)	0.003	0.374
Urban	1940 (98.7)	209 711 (91.8)			1894 (98.7)	5570 (98.4)		
Geographic location								
South	1178 (59.9)	115 918 (50.7)	0.090	<0.0001	1144 (59.6)	3203 (56.6)	0.025	0.029
Midwest	264 (13.4)	50 348 (22.0)	0.086		258 (13.4)	764 (13.5)	0.002	
Northeast	27 (1.4)	33 621 (14.7)	0.135		24 (1.3)	111 (2.0)	0.007	
West	496 (25.2)	28 654 (12.5)	0.131		493 (25.7)	1582 (28.0)	0.020	
Visit and clinical characteristics								
Charlson Comorbidity Index								
0	264 (13.4)	28 988 (12.7)	0.008	0.002	259 (13.5)	686 (12.1)	0.015	0.086
1–3	1407 (71.6)	158 216 (69.2)	0.025		1381 (72.0)	4218 (74.5)	0.027	
≥4	294 (15.0)	41 337 (18.1)	0.033		279 (14.5)	756 (13.4)	0.012	
Obesity	424 (21.6)	68 234 (29.9)	0.082	<0.0001	416 (21.7)	1202 (21.2)	0.007	0.684
Multiple grafts	1780 (90.6)	208 314 (91.1)	0.006	0.381	1741 (90.7)	5149 (91.0)	0.003	0.745
Ultrasonography used	221 (11.2)	2868 (1.3)	0.099	<0.0001	211 (11.0)	491 (8.7)	0.008	0.002
Robotic-assisted surgery used	15 (0.8)	1807 (0.8)	0.000	0.892	15 (0.8)	40 (0.7)	0.001	0.738

Categorical variables reported as count (%), and continuous variables as mean (SD) and median (interquartile range).

* Includes self-pay, workers’ compensation, other government payers, and other.

ASMD, absolute standardized mean difference; CABG, coronary artery bypass grafting; ICG, indocyanine green; IQR, interquartile range; NIRF, near-infrared fluorescence.

### Factors associated with all-cause emergency room use at 90 days postindex hospitalization

After controlling for covariates, a 16% lower all-cause ER use at 90 days postindex hospitalization was documented among the IFI group (Table 3). In the final multivariable model, several risk factors were independently associated with all-cause ER use at 90 days postindex hospitalization. The adjusted odds of 90-day ER use were 31% higher among black compared with White patients, 28% higher among females compared with males, and 19% lower among patients who did not self-identify as being either Hispanic or not Hispanic. The adjusted odds of 90-day ER use were 32% lower among patients with commercial indemnity health plans and 18% lower among patients with commercial managed care health plans compared with patients with Medicare. The adjusted odds of 90-day ER use were 34% higher among patients receiving care at hospitals in the Midwest compared with hospitals in the South. Patient clinical characteristics were also associated with 90-day ER use. The adjusted odds of 90-day ER use was 43% higher and almost three times greater among patients with 1–3 or 4 or more comorbidities, respectively. In addition, the adjusted odds of 90-day ER use were 26% higher among patients who were obese compared with nonobese patients (Table 3).

**Table 3:** Multivariable regression for assessing factors associated with 90-day postindex hospitalization all-cause emergency room use

		95% CI		
Predictor/covariate	aOR	Lower	Upper	*P*
NIRF with ICG				
NIRF with ICG (reference=no NIRF with ICG group)	0.84*	0.73	0.96	0.009
Patient demographics				
Sex				
Female (reference=male)	1.28	1.12	1.45	0.000
Age	1.00	0.99	1.00	0.249
Race				
White (reference)				
Black	1.31	1.01	1.69	0.033
Asian	1.29	0.82	1.97	0.268
Other/unknown	1.09	0.86	1.36	0.490
Ethnicity				
Hispanic or Latino	1.14	0.98	1.32	0.085
Not Hispanic or Latino (reference)				
Unknown	0.81	0.69	0.95	0.009
Payer type				
Medicare (reference)				
Medicaid	1.03	0.81	1.31	0.782
Commercial indemnity	0.68	0.48	0.94	0.026
Commercial managed care	0.82	0.68	0.97	0.039
Charity/indigent	0.72	0.45	1.14	0.154
Other	0.92	0.71	1.18	0.517
Hospital characteristics				
Hospital size (# of beds)				
1−299 beds	0.85	0.70	1.03	0.100
300−499 beds	1.13	0.98	1.31	0.106
500+ beds (reference)				
Teaching status				
Nonteaching hospital	0.95	0.82	1.11	0.560
Teaching hospital (reference)				
Population served				
Rural	1.51	0.98	2.29	0.053
Urban (reference)				
Geographic location				
South (reference)				
Midwest	1.34	1.12	1.61	0.002
Northeast	1.00	0.63	1.55	0.988
West	0.92	0.76	1.12	0.426
Visit and clinical characteristics				
Charlson Comorbidity Index categorized				
0 (reference)				
1–3	1.43	1.17	1.76	0.001
≥4	2.83	2.24	3.59	<0.0001
Obesity (reference=no)	1.26	1.09	1.44	0.001
Multiple grafts (reference=single)	0.88	0.72	1.09	0.238
Ultrasonography/TTFM used (reference=no)	0.99	0.81	1.20	0.912
Robotic-assisted surgery used (reference=no)	1.65	0.88	3.00	0.123

* Bootstrapped odds ratio for treatment group=0.843 (95% CI: 0.745–0.955).

aOR, adjusted odds ratio; CI, confidence interval; ICG, indocyanine green; NIRF, near-infrared fluorescence; TTFM, transit time flow measurement.

## Discussion

Our study suggests an association between the use of IFI and the reduction of all-cause ER visits at 90 days postindex hospitalization following CABG surgery compared with the comparison group. Ad hoc analyses found no differences in length of stay at index procedure, no patient count differences in percutaneous coronary intervention and percutaneous transluminal coronary angioplasty between the two groups within 90 days postindex hospitalization, and no differences between the two groups in postoperative mortality at 30 and 90 days. Another ad hoc analysis showed none of the patients assessed with IFI needed a repeat CABG procedure, whereas the comparison group experienced 35 repeat CABG procedures some of which involved multiple grafts within 90 days of postindex hospitalization. A potential reason for patients assessed with IFI not needing a repeat CABG procedure is that if a problem such as a deficit graft is discovered during the assessment with IFI, necessary corrections or revisions may be done at the index procedure. IFI is used to determine which grafts need to be revised as a quality assurance adjuvant, and all grafts that are found to be nonfunctional are presumably revised as is the standard of practice in cardiac surgery. Consequently, performing these unanticipated revisions at index procedure may lead to longer operative times.

Previous studies also support the use of IFI as an intraoperative graft patency assessment tool during CABG. A prospective evaluation of 348 coronary bypass grafts in 120 patients demonstrated that information from ICG angiograms led to graft revisions for technical problems in 4.2% of patients that would otherwise have been missed by the operating room team.^3^ A randomized trial comparing TTFM and IFI to conventional angiography in 106 CABG patients found that IFI was able to detect 83.3% of abnormal grafts, while TTFM only detected 25.0%, and angiography only detected 8.2% of grafts with at least 50% stenosis or completely occluded grafts.^2^ Another prospective study comparing IFI and TTFM in 100 CABG patients found the use of TTFM alone may lead to unnecessary graft revision, as IFI demonstrated adequate flow in some cases where TTFM indicated poor flow in 3.8% of grafts.^4^

We used ER use postdischarge instead of readmissions as a primary endpoint because visits to the ER are rising^28^ and frequent following CABG surgery.^16^,^17^ The Hospital Readmissions Reduction Program is a Medicare value-based purchasing program that many financially penalize hospitals when Medicare patients have above-expected rates of 30-day risk-standardized unplanned readmissions from the index admission for six conditions or procedures including CABG.^29^ It is reported that nearly half of hospitals would be categorized in a different performance category in comparison to their peers if ER visits were included.^16^ This means including ER visits could substantially change a hospital’s perceived performance.^16^ Thus, the Centers for Medicare and Medicaid Services (CMS) may achieve a more holistic view of a hospital’s quality of care if ER use was considered in addition to readmission rates when assessing a hospital’s performance.

In this study, we did not assess care cost differences between the treatment and comparison groups. However, previous studies found the use of IFI has been associated with cost reductions in hospital costs. Results from 350 patients undergoing CABG with IFI enrolled in the VICTORIA Multicenter Registry documented complication rates, including reoperation and length of stay, that were 50% lower than expected compared with similar patients enrolled in the Society of Thoracic Surgeons’ National Cardiac Database, one of the longest standing and largest existing medical data sets that exist today.^6^,^8^ A cost analysis performed independently by CMS also concluded that the use of IFI in CABG resulted in average cost reductions of $2000–$4000 per patient.^8^,^9^ The Sentara Heart Hospital study of 700 patients undergoing CABG also demonstrated that total costs of CABG were 4.2% lower and average length of stay was 6–16% lower in the 358 patients where the CABG procedure included IFI versus the 225 cases performed without IFI.^8^

There are several limitations in this study. First, the PINC AI Healthcare Database is a hospital administrative database, which means the identification of conditions, procedures, and medications rely on the accuracy of the hospital-reported diagnosis and procedure codes and chargemaster descriptions. Second, less than 1% (n=1965) of patients were assessed with IFI within our sample. This figure may be underestimated as the ICD-10-PCS code which may be used to report the use of ICG is often underreported by facilities. Therefore, the actual use of IFI may be higher than what was captured. We attempted to address this issue by also using chargemaster data and National Drug Codes to capture ICG use not reported with the appropriate ICD-10-PCS code. Third, we did not account for differences between the two groups regarding whether the left internal mammary artery or other arterial grafts were used. The most important and commonly used arterial graft is the left internal mammary artery due to its increased patency rate and resistance to atherosclerosis.^30^ We presume that most CABG patients received a left internal mammary artery, as it is considered the standard of care worldwide.^31^

## Conclusion

Our findings support that the use of intraoperative graft patency techniques such as IFI may improve the quality of CABG surgery and support CMS’ objective of improving healthcare. CABG patients intraoperatively assessed with IFI experienced lower all-cause ER use within 90 days postindex hospitalization compared with those not assessed with IFI. Routine intraoperative graft patency assessment with IFI may help to improve a patient’s care experience following index hospitalization as its use is associated with a reduction in subsequent resource utilization. Further studies are needed to compare the ER usage among centers that used this technique versus those that did not to determine if associated reductions in ER use are a center or technique-specific phenomenon.

## Provenance and peer review

Not commissioned, externally peer-reviewed.

## Ethical approval

Based on US Title 45 Code of Federal Regulations, Part 46, the study was exempted from institutional review board approval. The study consisted of records-based research and as such was exempt from ethics committee review and was Health Insurance Portability and Accountability Act-compliant.

## Sources of funding

Stryker Corporation provided funding for this study.

## Authors’ contribution

M.P.S.: conceptualization, writing – original draft, writing – review and editing, visualization, funding acquisition. D.G.N.: conceptualization, writing – review and editing. A.B.B.: writing – original draft, writing – review and editing, visualization. S.E.: conceptualization, methodology, formal analysis, investigation, data curation, writing – original draft, writing – review and editing. Z.C.: methodology, formal analysis, investigation. C.L.: conceptualization, data curation, methodology, formal analysis, investigation. V.A.: data curation, formal analysis, investigation. D.D.M.: conceptualization, writing – original draft, writing – review and editing.

## Conflicts of interest disclosure

M.P.S., D.G.M., and A.B.B.: Stryker Corporation employs. S.E., Z.C., C.L., and V.A.: Premier Healthcare employs. D.D.M. is a consultant to Stryker Corporation.

## Research registration unique identifying number (UIN)

Not aplicable.

## Guarantor

Michelle P. Sosa and Seth Emont.

## Supplementary Material
